# Molecular Interplay Between Non-Coding RNAs and Connexins and Its Possible Role in Cancer

**DOI:** 10.3390/ijms26062538

**Published:** 2025-03-12

**Authors:** Pablo Pérez-Moreno, Juan P. Muñoz, Mauricio A. Retamal

**Affiliations:** 1 Programa de Comunicación Celular en Cáncer, Instituto de Ciencias e Innovación en Medicina (ICIM), Facultad de Medicina Clínica Alemana, Universidad del Desarrollo, Santiago 7780272, Chile; 2 Laboratorio de Bioquímica, Departamento de Química, Facultad de Ciencias, Universidad de Tarapacá, Arica 1000007, Chile; jpmunozb@academicos.uta.cl

**Keywords:** non-coding RNAs, connexins, cancer

## Abstract

Non-coding RNAs (ncRNAs) are sequences that do not encode for proteins and play key roles in different cellular processes, including cell proliferation and differentiation. On the other hand, connexins (Cxs) are transmembrane proteins that principally allow intercellular communication. In pathological conditions such as cancer, there is a deregulation in the expression and/or function of ncRNAs and Cxs, which in turn leads to an enhancement in the aggressive phenotype, such as a greater proliferative and invasive capacity. This suggests a plausible interplay between ncRNAs and Cxs. Based on that, this review aims to summarize the current knowledge regarding this relationship and to analyze how it may influence the development of aggressive traits in cancer cells and the clinicopathological features of cancer patients. Finally, we discuss the potential of ncRNAs and Cxs as promising clinical biomarkers for cancer diagnosis, prognosis, and therapeutic targeting.

## 1. Introduction

Over the past century, a substantial amount of research has focused on mRNA-coding genes and their roles in cellular processes. However, a distinct class of molecules, known as non-coding RNAs (ncRNAs), has emerged as important players in cell biology because of their pivotal involvement in a wide array of cellular processes [1]. Once regarded as “junk RNA”, ncRNAs are now recognized as essential regulators of gene expression and cellular activity [2]. This diverse group of ncRNAs includes microRNAs (miRNAs), small nuclear RNAs (snRNAs), small nucleolar RNAs (snoRNAs), ribosomal RNAs (rRNAs), small interfering RNAs (siRNAs), PIWI-interacting RNAs (piRNAs), circular RNAs (circRNAs), and long non-coding RNAs (lncRNAs). Despite their highly variable sizes and structures, a defining characteristic of ncRNAs is their lack of protein-coding potential [3].

Beyond their structural diversity, ncRNAs interact with RNA, DNA, and proteins to regulate several biological processes, including post-transcriptional regulation, chromatin remodeling, transcriptional regulation, and acting as molecular scaffolds. These interactions are vital for maintaining cellular homeostasis. However, in cancer, the regulatory functions of ncRNAs are often disrupted, leading to tumor initiation, progression, and resistance to therapy [4].

Connexins (Cxs) are a family of transmembrane proteins that form specialized channels known as gap junction channels (GJCs), which enable direct communication between adjacent cells. These structures facilitate the exchange of ions, metabolites, and other small molecules, which is essential for maintaining cellular homeostasis and tissue coordination. Their function has been associated with cell growth, cell differentiation, and apoptosis [5,6]. In addition to forming GJCs, Cxs also form hemichannels at the non-junctional plasma membrane, which allow the exchange of ions and molecules, such as ATP and glutamate, between the cytoplasm and the extracellular space [7]. This dual functionality highlights their versatile roles in both intercellular and intracellular signaling.

The involvement of ncRNAs and Cxs has been explored in various pathologies, including central nervous system disorders, neurodegenerative diseases, and cardiovascular conditions [8]. In cancer, ncRNAs and Cxs have been implicated with tumoral progression by actively participating in the acquisition of features associated with greater cancer aggressiveness, such as angiogenesis, invasion, and metastasis [1,9]. For instance, dysregulated ncRNAs can modulate the expression and function of Cxs, further contributing to the tumor microenvironment and cancer cell communication. Therefore, in this review, we attempt to gather all the existing information regarding the functional interaction between ncRNAs and Cxs in the context of cancer to identify novel research directions in this emerging field.

## 2. Selection of Articles

**Figure 1:**
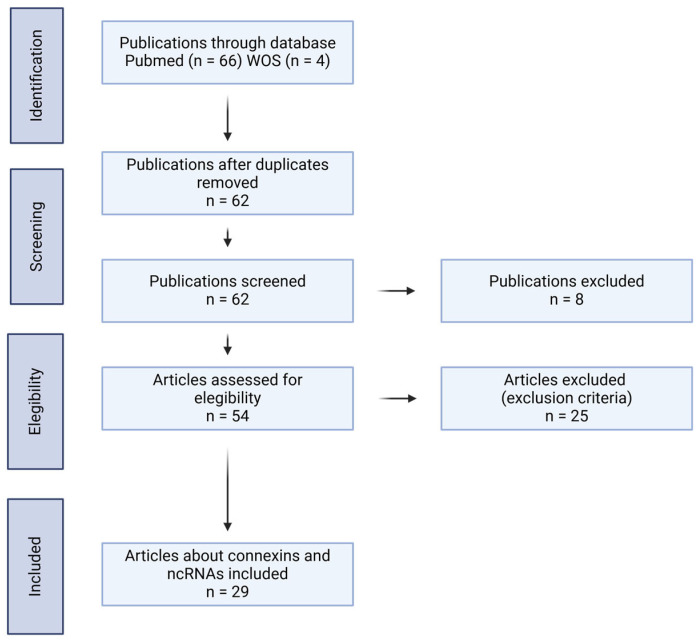
Methodology used to search for articles.

A search of the literature was performed by all authors using the Pubmed and Web of Science databases. The search terms were as follows: (ncRNAs OR non-coding RNAs OR miRNAs OR lncRNAs OR circRNAs or snRNAs OR snoRNAs OR siRNAs OR rRNAs OR piRNAs) AND (cancer OR carcinoma OR neoplasm) AND (connexins). The selection criteria were as follows: (1) interaction between ncRNAs and Cxs in cancer; (2) ncRNAs and Cxs related to aggressive phenotypic features in cancer; and (3) ncRNAs and Cxs expression detected in tissues and/or serum from cancer patients. The results not related directly to ncRNAs and Cxs in cancer were excluded from this review. The diagram used in this article is shown in Figure 1.

## 3. ncRNAs in Cancer

ncRNAs have emerged as critical regulators of gene expression and protein translation in cancer biology. Among them, miRNAs and lncRNAs are the most extensively studied [4].

**Figure 2:**
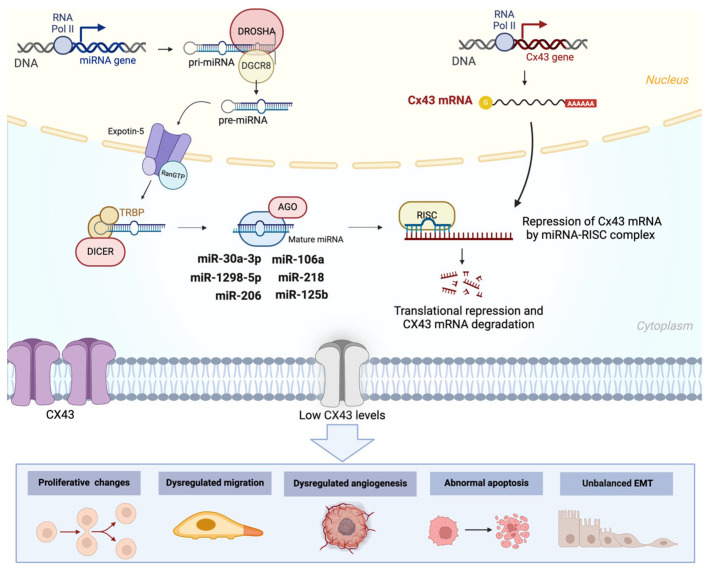
Regulation of Cx43 by miRNAs and its implications for cancer progression. miRNA biogenesis begins with the transcription of miRNA genes by RNA polymerase II, producing primary miRNAs (pri-miRNAs). These are processed in the nucleus by Drosha into precursor miRNAs (pre-miRNAs), which are then exported to the cytoplasm via Exportin-5 in a Ran-GTP-dependent manner. In the cytoplasm, DICER processes pre-miRNAs into mature miRNAs, which are incorporated into the RNA-induced silencing complex (RISC)—a multiprotein assembly (including Argonaute, AGO) that uses the mature miRNA as a guide to target complementary sequences in Cx43 mRNA. This interaction leads to translational repression and Cx43 mRNA degradation. Specific miRNAs, including miR-30a-3p, miR-1298-5p, miR-206, miR-125b, miR-218, and miR-106a, have been identified in different cancer types as regulators of Cx43 mRNA. Reduced Cx43 hemichannel levels result in dysregulated cellular functions critical to cancer progression, including proliferative changes, dysregulated cell migration, abnormal angiogenesis, apoptosis, and EMT dysregulation.

miRNAs are small ncRNAs that regulate gene expression post-transcriptionally by binding to complementary sequences in the 3′ untranslated regions (3′UTRs) of target mRNAs, leading to their degradation or translational repression. Their biosynthesis involves a multi-step process: primary miRNAs (pri-miRNAs) are transcribed by RNA polymerase II, processed in the nucleus by the Drosha–DGCR8 complex into precursor miRNAs (pre-miRNAs), and exported to the cytoplasm. There, Dicer processes pre-miRNAs into mature miRNAs, which are incorporated into the RNA-induced silencing complex (RISC) to execute their regulatory functions (Figure 2, upper panel) [10]. The miRNAs play a crucial role in maintaining cellular homeostasis and regulating processes, such as proliferation, differentiation, apoptosis, and immune responses. In cancer, this delicate balance is often disrupted, leading to the aberrant expression of miRNAs that function either as oncogenes or tumor suppressors. Oncogenic miRNAs promote tumorigenesis by downregulating tumor suppressor genes, while tumor-suppressive miRNAs inhibit cancer progression by targeting oncogenes [11]. For instance, miR-21 acts as an oncogene and is frequently overexpressed in tumor tissues of various cancers, including breast, prostate, and colon. In addition, it has been implicated in promoting the traits of cancer aggressiveness, such as chemoresistance and the acquisition of a cancer stem cell phenotype in cancer cells [12]. In contrast, miR-383 acts as a tumor suppressor, with a reduced expression observed in different cancers, including gastric, colorectal, hepatic, and pancreatic cancers. Furthermore, its overexpression reduces the acquisition of aggressive traits, such as proliferative, invasive, and metastatic capacity [13].

lncRNAs are RNA molecules exceeding 200 nucleotides in length that do not code for proteins but exert regulatory functions at transcriptional, post-transcriptional, and epigenetic levels. Their biosynthesis is similar to mRNAs; however, unlike mRNAs, lncRNAs often remain in the nucleus, where they interact with chromatin, transcription factors, or RNA-binding proteins to regulate gene expression. Some lncRNAs are also exported to the cytoplasm and modulate mRNA stability, translation, or protein localization [14].

lncRNAs also have a dual role in cancer, functioning either as oncogenes or tumor suppressors depending on their context and interactions. Oncogenic lncRNAs promote tumor progression by facilitating processes such as proliferation, invasion, metastasis, angiogenesis, and resistance to therapy. They achieve this through interactions with chromatin-modifying complexes, transcription factors, or other ncRNAs, often resulting in the activation of oncogenic pathways [15]. For example, LINC00662, which is highly expressed in cancer patients, such as breast, colon, and prostate [16], is notably overexpressed in gallbladder cancer, and their elevated levels are significantly associated with larger tumor size and lymph node metastasis. Mechanistically, LINC00662 promotes stemness, chemoresistance, invasion, and the epithelial–mesenchymal transition (EMT) via the LINC00662/miR-335-5p/OCT4 axis [17]. Another example is lncRNA CCAT1, which has been implicated in different neoplasms, including lung and cervical cancers [18]. In GBC, CCAT1 expression is notably elevated in tumor tissues, and its expression is significantly higher in advanced-stage tumors (T3 + T4) compared to early-stage tumors (T1 + T2). In addition, a high correlation exists between CCAT1 expression, lymph node metastasis, and advanced TNM stages, indicating its potential role as a marker of poor prognosis in GBC [19].

Conversely, tumor-suppressive lncRNAs inhibit cancer progression by maintaining genomic stability, repressing oncogenic signaling, and inducing apoptosis or senescence [20]. For instance, lncRNA MEG3, which has been described in multiple types of cancers, plays an antitumor role by promoting apoptosis and inhibiting angiogenesis through different signaling pathways [21].

circRNAs are a class of non-coding RNAs characterized by their covalently closed-loop structure, which distinguishes them from linear RNAs. These RNAs have gained attention for their regulatory roles in gene expression, splicing, and cellular processes. Emerging evidence suggests that circRNAs play important roles in various diseases, including cancer, by modulating key signaling pathways and cellular functions [22]. An example of this is circ-FOXO3, which was found to have decreased expression in tumor cell lines and tumor tissues compared to adjacent tissues. Furthermore, the overexpression of circ-FOXO3 was shown to decrease cell survival, promote cell apoptosis, and form smaller tumors in vivo [23].

piRNAs are small sequences between 24 and 31 nucleotides and are processed from single-stranded RNA precursors transcribed from intergenic regions by a Dicer-independent mechanism [24]. Research on piRNAs and their role in tumor progression has been steadily increasing in several types of cancers, including colorectal, gastric, liver, and gallbladder cancer [25]. An example of this is piR-823, which was found to be significantly upregulated in colorectal cancer tissues compared to nontumor tissues. An increased expression of piR-823 correlated with poorly differentiated tumors. The inhibition of piR-823 led to a reduction in cell proliferation and colony formation, induced cell cycle arrest in the G1 phase, and triggered apoptosis in CRC cell lines [26].

snoRNAs are non-coding RNAs that accumulate mainly in the nucleoli and are primarily responsible for the post-transcriptional modification and maturation of ribosomal RNAs, rRNAs, snRNAs, and other cellular RNAs. Interestingly, mutations and aberrant expression of snoRNAs have been reported in cancer, suggesting a potential role of snoRNAs as potential biomarkers and therapeutic targets [27]. An example is SNORD47, which was found to be downregulated in glioma tissue samples and inversely correlated with advanced tumor stage and overall survival. Notably, SNORD47 inhibits glioma cell proliferation by inducing G2 phase arrest. Furthermore, its overexpression suppresses both invasion and the epithelial–mesenchymal transition (EMT) in glioma cells, highlighting its role as a tumor suppressor [28].

snRNAs have been studied widely and are essential components of the spliceosome. They regulate gene expression by ensuring accurate mRNA maturation, and their dysregulation has been implicated in various cancers. Mutations or altered expression of snRNAs can lead to aberrant splicing events that promote tumorigenesis by affecting oncogenes and tumor suppressors. Emerging evidence suggests that specific snRNAs contribute to cancer progression, metastasis, and therapy resistance [29]. An example is U1 snRNP (U1), the most abundant small nuclear RNA (snRNA) in vertebrates, which suppresses proximal polyadenylation signals (PASs) in mRNAs. The inhibition of U1 has been shown to enhance migration and invasion of HeLa tumor cells in vitro, whereas its overexpression exerts the opposite effect [30]. Another snRNA, RNU5E-1, shows significantly lower expression levels in hepatocellular carcinoma (HCC) tissue compared to adjacent liver tissue. Moreover, reduced RNU5E-1 expression is strongly associated with larger tumor size, poor differentiation, advanced TNM stage, and worse overall survival in HCC patients [31].

rRNAs are essential for protein synthesis, and their deregulation has been linked to tumor progression. Alterations in rRNA biogenesis, modification, and processing can drive uncontrolled cell proliferation and therapy resistance [32]. One of the most abundant rRNA modifications involves the conversion of uridine to pseudouridine (Ψ) by pseudouridine synthases. A notable example is a cancer-specific single-nucleotide variation in 18S rRNA found in cancer patients, which disrupts proper mRNA processing and may promote aggressive cancer cell traits [33].

siRNAs are short, double-stranded RNAs that regulate gene expression through the RNA interference (RNAi) pathway, leading to sequence-specific degradation of target mRNAs. In cancer, siRNAs play a dual role, functioning as both natural regulators of oncogenic pathways and as potential therapeutic tools. The dysregulation of endogenous siRNA-like molecules can contribute to tumor progression by altering the expression of key oncogenes and tumor suppressors [34]. An example of this is the use of siRNAs targeting P53 mutants, which induce cell death and inhibit tumor growth, thereby delaying progression in xenograft models [35].

In summary, ncRNAs are fundamental in cancer biology, functioning as key regulators of gene expression and cellular processes. Their dual roles as oncogenes or tumor suppressors highlight their complex involvement in tumor progression, invasion, metastasis, and therapeutic resistance.

## 4. Connexins in Cancer

Cxs are ubiquitously expressed in nearly all tissues, and their function is crucial for processes such as embryonic development, tissue repair, and immune responses [6]. The dysregulation of Cxs expression has been implicated in numerous pathological conditions, including cancer, where they exhibit context-dependent behavior, acting as either tumor suppressors or oncogenes [9]. Thus, in some contexts, Cxs can suppress tumor initiation and growth, while in others, they contribute to tumor progression [36] and enhance metastatic potential [37]. For instance, Cx43 is one of the most studied Cxs, and it is known for its tumor suppressive properties in various types of cancers, including breast and colorectal cancer. Reduced expression of Cx43 correlates with increased cancer aggressiveness, such as higher proliferation rates and cell viability [36,38,39,40]. However, Cx43 has also been found to promote breast, prostate, and brain cancer metastasis under certain conditions [41,42,43,44]. Similarly, Cx46 has been shown to enhance aggressive traits in breast cancer cells, increasing migratory and invasive capacities and elevating the expression of genes associated with the epithelial–mesenchymal transition (EMT) and stem-like phenotypes, which are critical in cancer progression and metastasis [45]. Another example is Cx26, whose expression is elevated in CSC populations, which forms complexes with NANOG, promoting self-renewal in triple-negative breast cancers [46].

Despite these findings, the molecular mechanisms by which different Cxs exert their functions in various cancer cell types remain poorly understood. However, it is well established that Cxs form hemichannels that release ATP [47,48,49], along with other signaling molecules [50]. Within the tumor microenvironment, these hemichannels are likely to play an active role in angiogenesis, immune evasion, and therapy resistance, as ATP is a key regulator of these processes [51,52,53]. Notably, not all Cx hemichannels exhibit the same permeability to large molecules [54], suggesting that the impact of hemichannels in cancer progression depends on the specific Cx type forming the hemichannel and/or the regulatory mechanisms controlling its function within a particular tumor. Factors such as pH, redox potential, phosphorylation, and other post-translational modifications influence the opening and closing of these hemichannels [55,56,57]. Beyond their role as hemichannels, Cxs at the plasma membrane can also form direct protein-to-protein interactions with key cancer-related proteins such as Akt1 and Src, modulating their activity [58,59]. Moreover, the function of Cxs in cancer is influenced by their localization, as their role varies depending on whether they are situated in the plasma membrane or within intracellular compartments, such as the nucleus, regulating the gene expression of key proteins and transcription factors that ultimately impact cancer cell behavior [60,61]. In this context, recent studies have identified the presence of small fragments of Cx43, known as GJA1-11k, generated through an IRES-dependent mechanism. These fragments can localize to the nucleus of HEK-293 FT cells, suppressing cell growth by limiting cell cycle progression from the G_0_/G_1_ phase to the S phase [62]. In conclusion, the role of Cxs in cancer is influenced not only by their expression levels but also by factors such as Cx type, hemichannel functionality and permeability, protein–protein interactions, intracellular localization, and, as proposed in this review, their interactions with lncRNAs. Collectively, this evidence underscores the complexity of Cxs’ involvement in cancer biology and the need for further investigation.

Figure 2 shows a general overview of the effects of Cx43 modulation by miRNAs.

## 5. Interplay Between ncRNAs and Cxs in Cancer

### 5.1. Pancreatic Cancer

Pancreatic cancer, particularly pancreatic ductal adenocarcinoma (PDAC), is one of the most aggressive malignancies, characterized by a poor prognosis and limited treatment options [63]. The dense tumor microenvironment, hypoxia, and high metastatic potential are key factors contributing to its lethality. Recent research has highlighted the role of ncRNAs and Cxs in regulating tumor behavior and therapy resistance in PDAC [64].

In pancreatic cancer, miR-30b-5p was shown to be enriched in exosomes derived from PDAC cells. Additionally, exosomes from hypoxic PDAC cells were found to induce angiogenesis, which was mediated by the downregulation of Cx43. This suggests that the increase in miR-30b-5p and the decrease in Cx43 are linked to this effect. Moreover, it was demonstrated that the overexpression of miR-30b-5p significantly downregulated Cx43 (*GJA1*) gene expression, and this interaction was directly confirmed through a luciferase assay [65]. Similarly, it was shown that cells transfected with miR-30a-3p reduced tumor size in xenografts. Additionally, the inhibition of miR-30a-3p significantly increased gemcitabine cytotoxicity compared to controls. Interestingly, tumors transfected with miR-30a-3p displayed a significantly lower Cx43 expression, suggesting that miR-30a-3p inhibition could enhance chemotherapy responsiveness in PDAC xenografts through the upregulation of Cx43 [66]. In pancreatic cancer cells, genomic analyses revealed that the expression of four miRNAs was significantly correlated with prognosis: hsa-miR-30d-5p was linked to a favorable prognosis, while hsa-miR-8089, hsa-miR-146a-5p, and hsa-miR-21-3p were correlated with an unfavorable prognosis. Interestingly, a correlation analysis showed that a higher expression of hsa-miR-30d-5p was significantly associated with lower Cx43 expression. The hsa-miR-30d-5p/Cx43 axis was identified as a key potentially involved in pancreatic cancer metastasis [67]. These findings suggest the potential of targeting miRNA-Cx43 interactions as a novel therapeutic strategy in PDAC, particularly to counteract resistance to conventional therapies.

### 5.2. Bladder Cancer

Bladder cancer is one of the most common urological malignancies worldwide, with a high recurrence rate and significant morbidity. It encompasses a spectrum of diseases ranging from superficial, noninvasive tumors to highly aggressive, muscle-invasive bladder cancer (MIBC) [68].

In bladder tumors, miR-1298-5p was found to have reduced expression compared to normal tissues. In contrast, Cx43 levels were increased in bladder cancer patient tissues. Furthermore, the overexpression of miR-1298-5p inhibited cell proliferation, migration, and invasiveness in bladder cancer cell lines. Interestingly, this effect is mediated by miR-1298, which targets the 3′-UTR of Cx43, leading to a reduction in its expression [69]. Another miRNA involved in bladder cancer, miR-139-5p, exhibits reduced expression in both bladder cancer tissues and cell lines. The overexpression of miR-139-5p was found to reduce proliferation, migration, and invasion in bladder cancer cell lines. Interestingly, miR-139-5p was shown to directly bind Cx43 mRNA, resulting in decreased Cx43 mRNA and protein levels [70].

### 5.3. Breast Cancer

Breast cancer (BC) is one of the most prevalent malignancies worldwide and a leading cause of cancer-related deaths among women. It is a highly heterogeneous disease with diverse molecular subtypes, each characterized by distinct genetic and epigenetic alterations that influence tumor progression [71]. Cxs, particularly Cx43, have been implicated in both tumor-suppressive and tumor-promoting functions in BC. In this context, miR-206 was identified as a key regulator of Cx43 expression through a direct interaction with its mRNA. The overexpression of miR-206 was shown to decrease cell viability, proliferation, migration, and invasion in BC cell lines [72]. Additionally, miR-206 expression was positively correlated with lymph node metastasis in BC patients, suggesting that the miR-206/Cx43 axis could be a promising target for further study in BC treatment and progression. Interestingly, Cx43 expression was found to be significantly higher in lung and liver metastases compared to primary tumors in BC patients [72,73]. Similarly, miR-381 suppresses the expression of both Cx43 and C/EBPα (CCAAT/enhancer-binding protein α), inhibiting migration and invasion in BC cell lines. Additionally, decreased levels of miR-381 and increased expression of C/EBPα and Cx43 were observed in BC tissues and cell lines [74]. Another miRNA of interest is miR-200a, which directly targets the 3′-untranslated region (3′-UTR) of Cx43. Studies have shown that miR-200a expression is downregulated, whereas Cx43 expression is upregulated in metastatic breast cancer compared with primary tissue, respectively. Furthermore, the overexpression of Cx43 in BC cell lines was associated with enhanced migratory potential [75]. Surprisingly, the carboxy-terminal domain of Cx43 (CT-Cx43) has an important role in BC. The downregulation of CT-Cx43 and upregulation of miR-125b in low-grade human BC samples were observed. In vitro, CT-Cx43 overexpression resulted in decreased proliferation of MCF-7 cells. These data suggest that the CT domain of Cx43 is probably responsible for Cx43 function in BC [76].

lncRNA-CCRR was observed to be overexpressed in BC patients with brain metastases and even more highly expressed in patients with both brain and other metastases compared to those without metastasis. This suggests that a higher expression of lncRNA-CCRR may be associated with increased tumor aggressiveness. Additionally, it was observed that the overexpression of lncRNA-CCRR elevated Cx43 levels, and when siRNA against lncRNA-CCRR was used, Cx43 expression decreased, indicating that lncRNA-CCRR modulates Cx43 expression. Interestingly, the overexpression of lncRNA-CCRR increased dye transfer rates from astrocytes to MDA-MB-231BR/BT-474BR (brain metastatic variants of MDA-MB-231 and BT-474 cell lines, respectively) cells, but silencing lncRNA-CCRR suppressed MDA-MB-231BR/BT-474BR cell transmigration in a blood–brain barrier (BBB) model. This suggests that lncRNA-CCRR may promote communication between breast carcinoma cells and nontumor astrocytes [77].

### 5.4. Epithelial Ovarian Cancer

Epithelial ovarian cancer (EOC) is the most common and aggressive subtype of ovarian cancer, accounting for the majority of ovarian cancer-related deaths. Despite advances in treatment, including surgery and platinum-based chemotherapy, the overall survival rate remains poor, largely due to late-stage diagnosis, high recurrence rates, and the development of chemoresistance [71].

In EOC, miR-206 was found to be highly expressed in patients and correlated with a poor prognosis. The overexpression of miR-206 in EOC cell lines significantly enhanced migration and invasion, two hallmarks of cancer aggressiveness. Interestingly, miR-206 also increased cisplatin resistance by targeting Cx43, both in vitro and in vivo [78].

On the other hand, Cx26 is overexpressed in epithelial EOC cell lines and in patient tissues, and its overexpression is associated with a worse prognosis. Additionally, the overexpression of Cx26 promotes proliferation, migration, and invasion in vitro while also enhancing tumor growth in vivo. Interestingly, miR-2114-3p was identified as a direct regulator of Cx26 expression [79]. These findings suggest that the dysregulation of miRNA/Cx interactions, such as the miR-206/Cx43 and miR-2114-3p/Cx26 axes, may play crucial roles in EOC progression and chemoresistance.

### 5.5. Melanoma

Melanoma is a highly aggressive form of skin cancer that originates from melanocytes, the pigment-producing cells of the skin. While early-stage melanoma is often curable through surgical excision, advanced or metastatic melanoma has a poor prognosis because of its resistance to conventional therapies [80].

In melanoma cell lines, miR-335-5p was shown to reduce the expression of GJB5. Additionally, GJB5 expression was lower in metastatic melanoma lesions compared to primary lesions, suggesting a potential mechanism contributing to melanoma aggressiveness [81].

The reduced expression of Cx43 was observed in melanoma cell lines compared to human epidermal melanocytes. Furthermore, the overexpression of Cx43 was shown to inhibit cell proliferation and colony formation in vitro. Interestingly, miR-106a was shown to target the 3′ untranslated region (3′ UTR) of Cx43, thereby regulating its expression [82]. Furthermore, hypoxic conditions in the melanoma microenvironment increased the expression of miR-192-5p. This miRNA is transferred to cytotoxic T cells via Cx43, effectively inhibiting their antitumor activity. This finding points to a novel Cx-mediated immune evasion mechanism [83].

### 5.6. Glioblastoma

Glioblastoma multiforme (GBM) is the most aggressive and lethal form of primary brain cancer, characterized by rapid growth, extensive invasion into surrounding brain tissue, and resistance to standard therapies such as temozolomide (TMZ) and radiotherapy. The complex tumor microenvironment and the heterogeneous cellular composition of GBM contribute to its aggressiveness [84].

Cx43 expression plays a critical role in GBM cell invasion, and its impact depends on the cell type in which it is expressed. A study by McCutcheon et al. showed that when Cx43 expression is less in astrocytes, cell invasion decreases. However, when its expression is reduced in glioblastoma cells, the invasive capacity is restored. This suggests that the role of Cx43 in acquiring a more invasive phenotype depends on whether it is expressed in astrocytes or in tumor cells. Interestingly, miR-19b was identified as a direct regulator of Cx43, promoting increased migration and emphasizing the importance of the Cx43/miR-19b axis in GBM invasion [44].

miR-125b was also implicated in glioma progression, promoting glioma cell growth both in vitro and in vivo while inhibiting apoptosis by decreasing Cx43 expression [85]. Similarly, the miR-221/222 cluster directly targets Cx43, and its silencing reduces proliferation and migration in glioblastoma cell lines [86].

One notable function of Cxs in GBM is their ability to facilitate the transfer of miRNAs between cells, contributing to tumor aggressiveness [87,88]. In this regard, it was observed that the enhanced invasive capacity of glioma cells is partly promoted by the transfer of miR-5096 through Cxs from glioma cells to astrocytes, suggesting a new role for Cxs in the acquisition of aggressive traits in glioma cells [89]. Another example is miR-124-3p, which inhibits cell proliferation and tumor growth in glioblastoma cell lines in vivo. However, it achieves a greater inhibitory effect when transferred through GJCs to adjacent cells in glioblastoma cell lines [90].

In GBM, lncRNA NEAT1 is highly expressed in recurrent gliomas compared to primary gliomas. The downregulation of NEAT1 increases the sensitivity of GBM cell lines to temozolomide (TMZ). Additionally, NEAT1 acts as a competitive endogenous RNA for miR-454-3p. Interestingly, Cx43 was identified as a target of miR-454-3p, suggesting that elevated levels of NEAT1 and Cx43 contribute to chemoresistance to TMZ via a novel NEAT1/miR-454-3p/Cx43 axis [91].

In summary, the interplay between ncRNAs and Cxs in GBM highlights their significant roles in promoting tumor invasiveness, proliferation, and chemoresistance. Pathways such as the Cx43/miR-19b axis, miR-221/222 targeting of Cx43, and the NEAT1/miR-454-3p/Cx43 axis exemplify the molecular complexity driving GBM progression.

### 5.7. Other Types of Cancers

Recent studies indicate that ncRNAs play a significant role in the regulation of Cxs in other cancers. For instance, in colorectal cancer (CRC), miR-145-5p is transferred through Cxs, predominantly Cx43, between colorectal cancer cell lines in co-culture, inhibiting angiogenic capacity [92].

In nasopharyngeal carcinoma (NPC), miR-218 was found to be downregulated in both primary NPC tissues and cell lines. When miR-218 is overexpressed in NPC cell lines, it significantly reduces cell viability and induces apoptosis in vitro and also slows tumor growth in vivo. It was determined that one of the direct targets of miR-218 is GJA1 [93].

In gastric cancer (GC), decreased expression of Cx43 and increased expression of miR-301-3p were observed in both cell lines and tumor tissues from GC patients. Reduced Cx43 expression and elevated miR-301-3p levels correlate with poor differentiation, advanced TNM stage, vascular invasion, and lymph node metastasis. The overexpression of miR-301-3p promoted proliferation, migration, and invasion in GC cell lines. Interestingly, Cx43 mRNA is a direct target of miR-301-3p, suggesting that the observed effects could be mediated through the Cx43/miR-301-3p axis [94].

In prostate cancer (PC), it was observed that miR-20a is overexpressed in tumor tissue compared to normal nontumor tissue. Additionally, silencing miR-20a reduces proliferation in vitro and tumor growth in vivo. Interestingly, miR-20a binds directly to Cx43 mRNA, reducing its expression [95].

In choriocarcinoma (CCA), an increased expression of miR-935 was observed in both CCA cell lines and tumor tissues. The overexpression of miR-935 promoted cell proliferation, migration, invasion, tube formation, and tumorigenesis in vitro and in vivo. Interestingly, miR-935 directly targets Cx43, and it was found that the overexpression of METTL3 enhances miR-935 expression. This discovery highlights the METTL3/miR-935/Cx43 axis in CCA [96].

In HOS osteosarcoma cells, the overexpression of miR-23a reduces Cx43 levels, which delays osteoblast differentiation. In addition, it was observed that miR-23a binds directly to Cx43 mRNA by the luciferase assay. This suggests that Cx43 expression is directly associated with increased differentiation in osteosarcoma cells through the miR-23a/Cx43 axis [97].

Bioinformatic analyses have shown that the lncRNA LEF1-AS1 is overexpressed in tumor tissues of patients and is associated with worse survival outcomes in head and neck squamous cell carcinoma (HNSCC) patients. Moreover, it was demonstrated that LEF1-AS1 overexpression promotes proliferation, colony formation, increased resistance to apoptosis, migration, and invasion. It also induces the expression of genes associated with the epithelial–mesenchymal transition (EMT). Interestingly, LEF1-AS1 functions as a competitive endogenous RNA (ceRNA) by sponging miR-221-5p, leading to the overexpression of Cx43, which could further drive tumor progression [98].

A summary of the articles highlighting the interplay between ncRNAs and Cxs across all the previously discussed cancer types is provided in Table 1.

## 6. Potential Biomedical Applications of ncRNAs and Cxs

In recent years, ncRNAs have gained significant relevance in cancer research. Studies have shown that ncRNAs can promote aggressive traits in cancer cells, including enhanced migration, invasion, and chemoresistance. Furthermore, ncRNAs can serve as valuable biomarkers for prognosis, survival, and therapeutic response in cancer patients [1]. These advancements have significantly broadened the clinical applications of ncRNAs. Notably, ncRNAs can be detected in various biological samples, including tumor tissue, plasma, and serum, exhibiting high tissue specificity and sensitivity and demonstrating all the essential qualities required to serve as effective biomarkers in cancer patients [1,99]. For instance, MALAT-1 has been detected at elevated levels in plasma, showing a specificity of 84.8% in prostate cancer patients [100]. Similarly, HOTAIR has been identified in colorectal cancer patients, with a specificity of 92.5% [101]. Another example is miR-20, whose elevated expression has been observed in the early stages of non-small cell lung cancer (NSCLC) and positively correlated with TNM stage, positioning it as a promising biomarker for the early diagnosis of this malignancy [102]. In addition, LINC00662, which is overexpressed in various neoplasms, including breast and prostate [16], has shown an elevated expression in gallbladder cancer tissues and has been linked to the promotion of aggressive tumor cell traits. As expected, its heightened expression in tumor tissues correlates directly with increased lymph node metastasis and larger tumor size, underscoring its role in cancer progression [17]. All these data strengthen the idea that ncRNAs can be used as biomarkers of prognosis and/or progression in patients with cancer.

The use of ncRNAs in therapeutic applications has emerged as a promising avenue in modern medicine for the treatment of cancer [4]. As mentioned above, ncRNAs have demonstrated their potential to regulate gene expression at various levels, from transcription to translation. Furthermore, their ability to target multiple genes and pathways simultaneously offers a unique advantage over traditional therapies, thus offering a wide range of treatment possibilities [99]. For example, siRNA and CRISPR-based therapies can modulate ncRNAs expression, altering the malignant phenotype of cancer cells [103,104]. Importantly, the application of liposomal systems as delivery vehicles for inhibitory molecules (such as siRNAs) or activators (such as miRNA mimics) has emerged as a promising strategy in cancer therapy. Liposomal vehicles offer significant advantages, including efficient encapsulation of therapeutic molecules, enhanced stability, and ease of systemic administration, making them an attractive option for advancing ncRNA-based treatments [105,106]. An example of the therapeutic potential of ncRNAs is a study by Beg et al., in which MNX34, an miR-34a mimic which functions as a tumor suppressor, showed antitumor activity in a subset of patients with refractory advanced solid tumors in a phase 1 study [107]. These data reinforce the idea of using ncRNAs as therapeutic targets and pave the way for innovative treatment approaches in oncology.

Regarding Cxs, they have gained significant attention for their dual function in cancer, as they can act as oncogenes or tumor suppressors. Beyond their traditional role, Cxs are now recognized for their potential as therapeutic targets and biomarkers in clinical oncology because they are implicated in processes that drive tumor progression, including migration, invasion, proliferation, and drug resistance [36], as well as being used as clinicopathological biomarkers in different cancers [9]. For example, high levels of Cx43 have been associated with reduced progression-free survival (PFS) and recurrence-free survival in pTa and Pt1 bladder cancer patients [108]. On the other hand, Cx26 has been associated with high histological grade and worse prognosis in colorectal and BC patients [109,110], positioning Cxs as a very attractive group of proteins for their use as clinical biomarkers.

Additionally, Cxs offer a novel approach to disrupt or improve cancer cell communication depending on the tumoral context as the topic of treatment. Regarding this, modulators of Cx functions, such as peptides or bioactive compounds that block Cxs channels or allow intercellular communication, could be a potential strategy to limit tumor growth and cancer progression [111]. For instance, αCT1, a peptide designed against Cx43, promotes the formation of intercellular connections in HER2+ BC cells, promoting Cx43 stabilization, cell death, and decreased mammosphere formation capacity in vitro, demonstrating an antitumor effect in these cells [112].

Finally, it has been shown that Cxs can be detected in extracellular vesicles [113,114,115], which is a great indication that may allow the detection of these proteins in noninvasive samples such as serum, plasma, saliva, or urine, improving their use as biomarkers for prognosis, diagnosis, and treatment in cancer patients.

## 7. Conclusions

The currently available technological advances in molecular biology and bioinformatics have increased the possibilities of studying both direct and indirect interactions between proteins and ncRNAs. This review highlights 27 ncRNAs that demonstrate either direct or indirect interactions with Cxs. Of these ncRNAs, the majority were miRNAs and, to a lesser extent, lncRNAs. Other ncRNA types, such as circRNAs and piRNAs, have not yet been explored in the context of their interaction with Cxs, representing an opportunity for further research. Among the Cxs, Cx43 was predominantly described, and the principal modulation mechanism identified was the direct binding of miRNAs to Cx43 mRNA. This reflects its pivotal role in various cancers and its susceptibility to post-transcriptional regulation by miRNAs. In contrast, the mechanisms associated with lncRNAs primarily involve competitive endogenous RNA (ceRNA), a process that has been well-documented in the literature. Additionally, emerging evidence suggests that lncRNAs may also modulate Cx expression and function through epigenetic mechanisms, further adding complexity to this regulatory axis.

Mechanistically, Cxs exhibit dual roles as oncogenes or tumor suppressors in different cancers, showing a great variability between cancer types. This variability is influenced by tumor type, cellular context, and external microenvironmental factors, making the study of Cxs and their interactions particularly complex. Importantly, this review highlights the functional interactions between ncRNAs and Cxs as an underexplored research niche, offering significant opportunities for new investigations, particularly in elucidating context-specific regulatory mechanisms in different cancer types. Given their role in modulating gene expression and intercellular communication, these interactions could influence critical cancer hallmarks such as proliferation, apoptosis, and metastasis.

In recent years, liquid biopsy has been growing rapidly as a novel method for the detection of new clinical biomarkers. In this regard, integrating biomarkers like Cxs and ncRNAs into liquid biopsy platforms could revolutionize cancer diagnosis and monitoring. ncRNAs, due to their stability and ease of detection in various biological fluids, have shown great promise as clinical biomarkers for cancer progression and prognosis. On the other hand, Cxs, due to their protein nature, have a high potential as biomarkers. However, further studies are required to validate the use of Cxs through noninvasive methods. Moreover, considering the regulatory crosstalk between ncRNAs and Cxs, their combined analysis in liquid biopsy platforms could enhance diagnostic accuracy and provide a more comprehensive molecular landscape of tumors.

Finally, despite the growing evidence on the individual roles of ncRNAs and Cxs in cancer progression, their combined clinical potential remains largely unexplored. Current studies have primarily focused on their separate contributions as biomarkers and therapeutic targets, with limited research addressing their interplay in a translational context. Therefore, future clinical studies should prioritize investigating this axis as a unified entity rather than isolated components, potentially unveiling novel biomarker panels and therapeutic strategies that harness the synergistic effects of ncRNA–connexin interactions. Targeting lncRNA-Cx interactions may also offer new therapeutic avenues, including modulation of gap junction functionality or ncRNA expression to alter tumor behavior.

In summary, this review describes the intricate interactions between ncRNAs and Cxs and their potential clinical applications in cancer patients. These findings highlight an underexplored and promising field of study. 

## Figures and Tables

**Table 1:** Mechanisms associated with the ncRNA/connexin axis.

ncRNA	Cancer Type	Connexin-Associated Mechanism	Functional Effect	Clinicopathological Association	Reference
miR-30b-5p	Pancreatic Cancer	Binds directly to Cx43 mRNA	Increases angiogenesis	ND	[65]
miR-30a-3p	Pancreatic Cancer	Binds directly to Cx43 mRNA	Reduces tumor size	Highly expressed in cancer tissues and a negative correlation with disease staging and Cx43 expression	[66]
miR-30d-5p	Pancreatic Cancer	Inversely correlated with Cx43 expression	ND	Better prognosis	[67]
miR-1298-5p	Bladder Cancer	Binds directly to Cx43 mRNA	Inhibits invasion, proliferation, and invasion	Reduced expression in bladder cancer tissues	[69]
miR-139-5p	Bladder Cancer	Binds directly to Cx43 mRNA	Reduces proliferation, migration, and invasion	Reduced expression in bladder cancer tissues	[70]
miR-206	Breast Cancer	Binds directly to Cx43 mRNA	Decreases cell viability, proliferation, migration, and invasion	Reduced expression in BC tissues	[72,73]
	Ovarian Cancer	Binds directly to Cx43 mRNA	Increases invasion, migration, and cisplatin resistance	Poor prognosis and highly expressed in EOC tissues	[78]
miR-381	Breast Cancer	Suppresses C/EBPα-dependent Cx43 expression	Reduces migration and invasion	Reduced expression in BC tissues	[74]
miR-200a	Breast Cancer	Binds directly to Cx43 mRNA	Reduces migration	Downregulated in metastatic BC tissues	[75]
miR-125b	Breast Cancer	Inversely correlated with CT-Cx43 expression	Increases proliferation	Increased expression in BC tissues	[76]
lncRNA-CCRR	Breast Cancer	Directly correlated with Cx43 expression	Promotes communication between BC cells and astrocytes	Highly expressed in metastatic BC cancer tissues	[77]
miR-2114-3p	Ovarian Cancer	Binds directly to Cx26 mRNA, inhibiting the PI3k pathway	Inhibits tumor growth and invasion and induces S phase arrest of EOC cells	Reduced expression in EOC tissues and associated with better prognosis	[79]
miR-335-5p	Melanoma	Inversely correlated with GJB5 expression	ND	GJB5 underexpression is associated with overall worse survival	[81]
miR-106a	Melanoma	Binds directly to Cx43 mRNA	Increases proliferation and colony formation capacity	ND	[82]
miR-192-5p	Melanoma	Under hypoxic conditions, miR-192-5p is transferred to cytotoxic T cells via Cx43	Inhibits the antitumor activity of T cells	ND	[83]
miR-19b	Glioblastoma	Transfer of miR-19b Cx43-dependent from GBM cells to astrocytes	Increases invasion	ND	[44]
miR-125b	Glioblastoma	Binds directly to Cx43 mRNA	Inhibits apoptosis and promotes colony formation in vitro and tumor growth in vivo	ND	[85]
miR-221/222	Glioblastoma	Binds directly to Cx43 mRNA	Silencing of miR-221/222 reduces proliferation and migration capacities	ND	[86]
miR-5096	Glioblastoma	Transfer of miR-5096 Cx43-dependent from GBM cells to astrocytes	Silencing of miR-5096 reduces migration capacity	ND	[89]
miR-124-3p	Glioblastoma	Transfer of miR-124-3p Cx-dependent in GBM cells (Cx not specified)	Reduces proliferation, colony formation, and tumor growth in vivo	ND	[90]
NEAT1	Glioblastoma	Acts as a competitive endogenous RNA for miR-454-3p, increasing Cx43 levels	Increases resistance to TMZ	Highly expressed in recurrent gliomas compared to primary gliomas	[91]
miR-145-5p	Colorectal	Transfer of miR-145-5p Cx43-dependent through HMEC and CRC cells	Inhibits angiogenic process in vitro	ND	[92]
miR-218	Nasopharyngeal Cancer	Binds directly to Cx43 mRNA and regulates the SLIT/ROBO pathway	Reduces cell viability, apoptosis in vitro, and tumor growth in vivo	Downregulated in NPC tissues and cell lines	[93]
miR-301-3p	Gastric Cancer	Binds directly to Cx43 mRNA	Increases migration, invasion, and proliferation	Increased expression in cell lines and GC tissues and correlated with poor differentiation, advanced TNM stage, vascular invasion, and lymph node metastasis	[94]
miR-20a	Prostate Cancer	Binds directly to Cx43 mRNA	Silencing of miR-20a reduces proliferation in vitro and tumor growth in vivo	Overexpressed in tumor tissue	[95]
miR-935	Choriocarcinoma	Binds directly to Cx43 mRNA	Increases cell proliferation, migration, invasion, tube formation, and tumorigenesis in vitro and in vivo	Increased expression in CCA cell lines and tumor tissues	[96]
miR-23a	Osteosarcoma	Binds directly to Cx43 mRNA	Delays osteoblast differentiation	ND	[97]
LEF1-AS1	Head and Neck Squamous Cell Carcinoma	Competitive endogenous RNA (ceRNA) by sponging miR-221-5p, leading to the overexpression of Cx43	Overexpression promotes proliferation, colony formation, resistance to apoptosis, migration, EMT, and invasion	Overexpressed in tumor tissues and associated with worse survival	[98]

ND: not determined.

## Abbreviations

BC	breast cancer
CCA	choriocarcinoma
circRNA	circular RNA
CRC	colorectal cancer
EMT	epithelial–mesenchymal transition
EOC	epithelial ovarian cancer
GC	gastric cancer
GBM	glioblastoma multiforme
GJC	gap junction channel
HNSCC	head and neck squamous cell carcinoma
lncRNA	long non-coding RNA
miRNA	microRNA
MIBC	muscle-invasive bladder cancer
ncRNA	non-coding RNA
NEAT1	Nuclear-Enriched Abundant Transcript 1
NPC	nasopharyngeal carcinoma
PC	prostate cancer
PDAC	pancreatic ductal adenocarcinoma
piRNA	PIWI-interacting RNA
PFS	progression-free survival
rRNA	ribosomal RNA
siRNA	small interfering RNA
snoRNA	small nucleolar RNA
snRNA	small nuclear RNA
TNM	Tumor Node Metastasis
CCA	choriocarcinoma
circRNA	circular RNA
